# Overwork and Disease

**Published:** 1892-08

**Authors:** 


### OVERWORK AND DISEASE.

Overwork, whether of muscle or brain, is harmful and often fatal; but what is overwork for one man may be nothing but wholesome activity for another. Various causes may have lowered one's natural powers of endurance—lack of sleep, exhausting excitements, sedentary habits, an undue accumulation of fat, a weakened heart, or other organic disease. In all competitive sports it is dangerous for the contestants to ignore such physical differences. Spirit and excitement may help to win a temporary victory at too great a cost.

Most intelligent persons know that athletes are peculiarly liable to heart disease, and, as a class, are short-lived. It is well known, too, that exhausting marches, like the retreat of Napoleon's army from

Russia are attended by a frightful loss of life; but even the medical profession has not understood the nature of the relation between over-work and its morbid effects.

Of late years, however, the subject has been carefully studied by medical experts, and the general conclusion reached is that the system poisons itself by overwork and exhausting fatigue. The effect, in short is somewhat like what takes place when the eliminating organs of the body are debilitated or diseased, causing a retention of poisonous waste.

In the lower degrees of over-work, rest restores the system to its normal state by a speedy elimination of the injurious elements as poisons received from without are eliminated, and a fatal result avoided.

In more prolonged fatigue there is a rise of temperature and an alteration of the liquids of the body—a manifest feverish condition. In still more prolonged and severe exertions, there are changes in the bodily tissues, as well as in the fluids, especially in the heart and blood vessels, the kidneys and spinal cord. This is the case in forced marches, night watching followed by daily toil, in the persistent "cramming" of the schools, in the incessant drive of business, especially when these are associated with poor living and insufficient sleep. The Medical Journal says:

In some cases death occurs too soon for the development of the above symptoms. Thus the soldier fell dead after announcing the victory of Marathon. In Algeria two noted runners fell dead the instant they reached the goal. This sudden death from over-exertion is due to self poison by carbon doxide, which is formed more rapidly than the lungs can exhale it.

